# Treatment Modalities for Internet Addiction in Children and Adolescents: A Systematic Review of Randomized Controlled Trials (RCTs)

**DOI:** 10.3390/jcm12093345

**Published:** 2023-05-08

**Authors:** Shahana Ayub, Lakshit Jain, Shanli Parnia, Anil Bachu, Rabeea Farhan, Harendra Kumar, Amanda Sullivan, Saeed Ahmed

**Affiliations:** 1 Cornerstone Family Healthcare, Newburgh, NY 12550, USA; 2 Department of Psychiatry, University of Connecticut, 263 Farmington Avenue, Farmington, CT 06032, USA; 3 CIMPAR, 101 Madison St. Suite 300, Oak Park, IL 60302, USA; 4 Psychiatry Residency Program, Baptist Health UAMS Hospital, 3500 Springhill, Suite 100, North Little Rock, AR 72116, USA; 5 AHN Psychiatry and Behavioral Health Institute, 4 Allegheny Center, 8th Floor, Pittsburgh, PA 15212, USA; 6 Rutgers School of Public Health, 683 Hoes Ln W, Piscataway, NJ 08854, USA; 7 Dow University of Health Sciences Karachi, Karachi City 74200, Pakistan; 8 Quinnipiac University, 275 Mount Carmel Avenue, Hamden, CT 06518, USA; 9 Rutland Regional Medical Center, 160 Allen Street, Rutland, VT 05701, USA

**Keywords:** internet addiction, problematic internet use, children, adolescents, treatment, randomized controlled trial

## Abstract

Background: In recent years, the use of the internet among children and adolescents has dramatically increased, leading to growing concerns regarding the potential risks of excessive internet use and addiction. Addressing these concerns, this systematic review aims to summarize current evidence on the effectiveness of treatment interventions for internet addiction among children and adolescents. Method: We performed a systematic review using PubMed, Web of Science, PsycInfo, and Google Scholar with search terms including “internet addiction”, “problematic internet use”, “children” or “adolescents”, “treatment” and “randomized controlled trial”. We found 10 Randomized Controlled Trials (RCTs) meeting the criteria and included them in this systematic review. Results: This systematic review analyzed 10 randomized controlled trials focused on treatment interventions for internet addiction in adolescents and young adults. The interventions used were diverse, including cognitive-behavioral therapy (CBT), medication, electro-acupuncture (EA), and solution-focused approaches. The measures used to assess the effectiveness of the interventions also varied, but most studies reported moderate to large effect sizes for at least some outcomes. Overall, the studies suggest that interventions such as CBT and EA can be effective in reducing symptoms of internet addiction, internet gaming disorder, and unspecified internet use disorders. School-based programs and brief manualized CBT programs also show promise, though more research is needed to determine their long-term effectiveness. Conclusion: Promising treatment approaches for internet addiction are emerging, but inconsistencies in conceptualization, language, and diagnostic criteria present some challenges. The growing recognition of problematic internet use, as shown by the DSM-5′s recognition of Internet Gaming Disorder, highlights the need for a multidisciplinary approach and standardized criteria to facilitate accurate reporting across studies. Continued research is needed to identify effective treatments and diagnostic criteria for internet addiction, with the potential to offer practical insights into effective medications and therapies.

## 1. Introduction

The internet is an important tool for communication, education, work, and entertainment. In recent years, as the COVID-19 restrictions made it even more essential, the number of internet users worldwide rose from 738 million in 2000, to 4.9 billion in 2021; an increase of over 567% in just two decades [1]. According to Internet World Stats, the year 2019 witnessed a considerable increase in the number of individuals using the internet on a global scale. Approximately 4.5 billion individuals, accounting for 58.8% of the world’s population, were active internet users. This substantial proportion indicates that more than half of the global populace was engaged in online activities during this time period [2]. Affordable, accessible mobile technology has made it easier for people to access the internet, with 3.8 billion smartphone users worldwide as of 2021, up from just over 1 billion in 2013 [3]. This has resulted in many kids having early access to the internet; the American Community Survey (ACS) found that 95% of 3- to 18-year-olds in the US have home internet access. Of these children, 88 percent access the internet through a computer and 6 percent through a smartphone [4]. With this rise in number, there have been increasing concerns of internet addiction among the general public for the past two decades. In light of these concerns, researchers have sought to better understand and define the concept of internet addiction (IA), also known as problematic internet use (PIU) or Internet Addiction Disorder (IAD). These terms are often used interchangeably in literature. Some researchers might use “Internet Addiction Disorder” to emphasize the severity of the condition and its potential classification as a formal disorder, while “Internet Addiction” may be used more broadly to discuss the general phenomenon.

Internet addiction could be perceived as being more similar to a traditional substance addiction, characterized by a lack of control and withdrawal symptoms [5]. In contrast, Problematic Internet Use (PIU) encompasses a wider range of issues associated with internet usage, such as social, behavioral, and emotional challenges [6]. It is worth noting that some sources define PIU and IAD as a single entity, characterized by excessive or poorly controlled preoccupations, urges, or behaviors regarding computer use and internet access, resulting in impairment or distress [7]. In this paper, we will primarily use the term “Internet Addiction”, which is defined as a condition characterized by excessive or poorly controlled preoccupations, urges, or behaviors related to computer use and internet access, leading to impairment or distress [8].

Internet addiction has increasingly become a prevalent mental health issue especially among adolescents. Excessive internet use can have a serious impact on mental health. Some of the negative consequences include insomnia, anxiety, depression, low self-esteem, impulsiveness, mood disorders, poor family relationships, self-harm, suicidal ideation, suicide attempts, and even suicide [9,10,11]. Adolescent internet addiction is influenced by several factors. The widespread availability of mobile devices, such as smartphones, has facilitated a constant online presence among today’s younger generation. The internet serves as a means of communication for many individuals, and in some cases, it provides an avenue for escape from stress. However, when reliance on the internet becomes the primary coping mechanism for dealing with stress [12], it can lead to a range of negative consequences. 

First conceptualized as an impulse control disorder [13], there is now increasing evidence identifying internet addiction as a behavioral addiction [14,15]. This shift in understanding led the American Psychiatric Association (APA) to acknowledge that excessive gaming behavior, which falls under the umbrella of internet addiction, can be a source of clinical concern, and that research on this topic is ongoing. The World Health Organization (WHO) has officially included “Gaming Disorder” (GD) as a mental health condition in the latest (eleventh) revision of the International Classification of Diseases (ICD-11) [16]. The American Psychiatric Association (APA) has also addressed this issue by specifying internet addiction as internet gaming disorder (IGD) in Section III research criteria of the DSM-5 [17].

Researchers have used a range of various terms such as Digital Technologies Negative Use, problematic internet use, smartphone addiction, internet gaming disorder, internet gambling disorder, and others, which can lead to inconsistencies in data gathered and a lack of clarity in research findings [18]. Due to lack of clear terminology, different definitions, and diagnostic criteria for internet addiction can make it difficult to accurately estimate prevalence rates among different populations and time periods. The studies report rates as varied as 1.6% for internet gaming disorder in Europe, to 50% in Korea [19].

The growing prevalence of internet addiction among children and adolescents suggests that more children and adolescents are at risk of developing negative mental health consequences. To better understand how internet addiction and mental health are connected, scientists have used brain-imaging methods to investigate the impact on internet addiction on the brain. Numerous research studies have underscored the impact of excessive internet use on the brain’s structure and function. These studies reported alterations in various brain regions, such as frontal lobe, temporal lobe, limbic system, cerebral blood flow, a reduction in both gray matter volume and white matter integrity, particularly in the corpus callosum [20,21,22,23,24,25]. Such changes have been associated with a decline in executive functioning, cognitive abilities, attentional capacity, and language development [26], further emphasizing the need for moderation in internet usage [26,27,28] Excessive internet use may affect the reward system of the brain, which is responsible for the release of dopamine, and lead to desensitization and tolerance, which can contribute to addiction. There is evidence, which suggests that internet addiction can activate the same reward pathways in the brain as non-behavioral drug addictions [29,30,31] like opioids and alcohol. Functional magnetic resonance imaging (fMRI) studies have shown altered neural responses in the reward-related regions of the brain in individuals with internet addiction, including the prefrontal cortex, anterior cingulate cortex, and striatum [21,29,30,32,33].

The above studies have demonstrated neurobiological changes in the brains of individuals with Internet Addiction (IA), which show similarities to those observed in individuals with substance use disorders (SUDs) [31,32,33,34,35]. Drug addiction studies reveal that relapse vulnerability arises from intense desires and diminished control over drug-seeking behavior [36]. Brain changes, especially in the prefrontal cortex, contribute to compulsive drug-taking and disadvantageous behaviors [37]. Cocaine-dependent individuals exhibit decreased gray matter concentration in brain regions involved in decision-making, behavioral inhibition, and emotional processing [38]. Similarly, heroin-dependent individuals show reduced gray matter density in the prefrontal, temporal, and cingulate cortices, with duration of use negatively correlating with gray matter density [39]. These findings parallel the neurobiological changes observed in internet addiction. These similarities in neuroimaging findings indicate that IA and SUD share common neurobiological mechanisms. Understanding the neurobiological mechanisms underlying internet addiction in children and adolescents can help inform the development of effective interventions and treatments. The complexity of the condition, including alterations in brain structure, function, neurotransmitter systems, and genetic factors, highlights the need for a multidisciplinary approach. 

Given the sensitive nature of internet addiction, particularly among children and adolescents, it is essential to approach this topic with caution and ensure that readers are provided with accurate and balanced information. Several studies have explored potential interventions for internet addiction, including psychosocial therapies, pharmacological treatments, and complementary medicine techniques [40,41,42,43,44]. It is important to recognize the potential risks, limitations, and controversies surrounding some of these interventions, particularly when considering the use of psychotropic medications [45] or complementary medicine techniques like electro-acupuncture [46]. To ensure a careful and cautious approach, our focus will primarily be on the cognitive mechanisms responsible for internet addiction, while acknowledging the need for further research and validation of various intervention methods.

The aim of this systematic review is to synthesize and evaluate the current research on interventions for internet addiction in adolescents and young adults, with a focus on randomized controlled trials. By examining the efficacy of various interventions and identifying gaps in the current literature, this review aims to provide valuable insights for future research and clinical practice.

## 2. Materials and Methods

This systematic review followed the Preferred Reporting Items for Systematic Reviews and Meta-Analyses (PRISMA) guidelines.

### 2.1. Search Strategy

**Figure 1:**
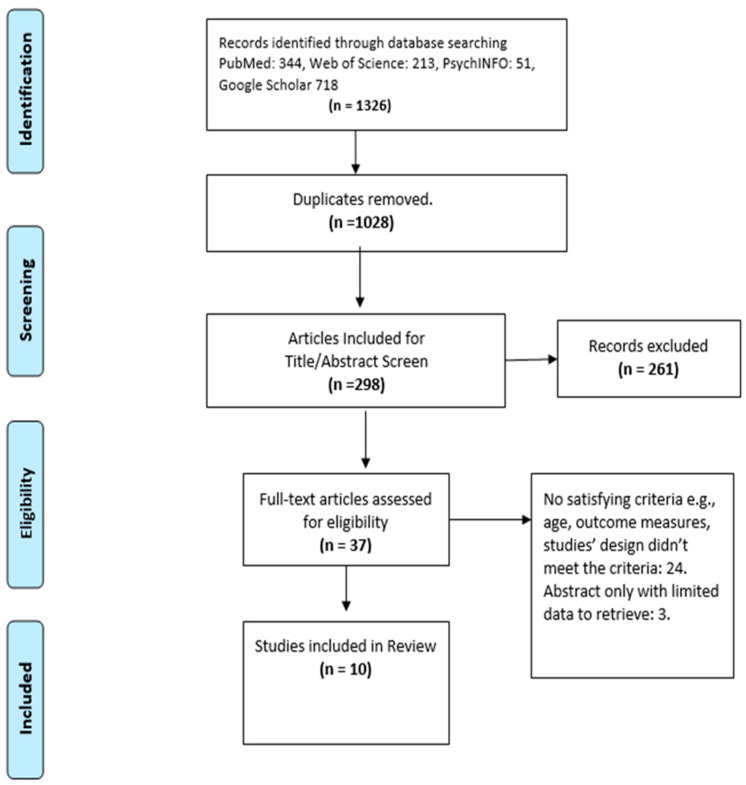
PRISMA Flow Diagram.

A comprehensive literature search was conducted using four electronic databases, including PubMed, Web of Science, PsychInfo, and Google Scholar. The following keywords and mesh terms were used: “internet addiction” OR “problematic internet use” AND “children” OR “adolescents” AND “treatment” AND “randomized controlled trial”. The search was limited to studies published in English language within the past 15 years, between 2008 and 2023. As shown in Figure 1, on initial search, we initially found 1326 studies. After removing duplicates, we had 1028 studies left. Two separate reviewers (SP and SA) carefully looked at the titles and abstracts of 298 studies. They accepted 37 full-text articles for closer examination. After reviewing these articles, we included 10 studies in our systematic review.

### 2.2. Inclusion Criteria

The following inclusion criteria were applied: randomized controlled trials (RCTs) that focused on the treatment of internet addiction in children and adolescents (ages 21 and under). Studies reporting on any type of intervention (e.g., medication, psychotherapy, combined treatments) were included. Studies that included mixed populations but reported results for the subgroup of children and adolescents were also included. In this study we use “internet addiction” to represent the interchangeable terms usually described in previous studies such as internet addiction, internet use disorder, problematic online gaming, internet addiction disorder, internet gaming disorder, or problematic internet use.

### 2.3. Exclusion Criteria

Case reports or case series, studies not focused on children and adolescents, they were not designed as randomized controlled trials, or if they did not concentrate on the treatment of internet addiction.

### 2.4. Study Selection

Two independent reviewers (SP and SA (Saeed Ahmed)) screened the titles and abstracts of the identified articles to determine their eligibility for inclusion. Full-text articles of the potentially relevant studies were then assessed for eligibility, and data were extracted using a standardized form. Any discrepancies between the reviewers were resolved by a third reviewer (LJ) through discussion and consensus.

### 2.5. Quality Assessment

The risk of bias of each included study was assessed using the Cochrane Risk of Bias tool. This tool assesses the quality of each study based on seven domains: random sequence generation, allocation concealment, blinding of participants and personnel, blinding of outcome assessment, incomplete outcome data, selective reporting, and other biases. The risk of bias for each domain was rated as low, unclear, or high.

### 2.6. Data Synthesis and Analysis

Data was synthesized using a narrative approach. The main characteristics and findings of the included studies were summarized in Table 1. A meta-analysis was not performed due to heterogeneity in the interventions and outcomes reported in the included studies.

## 3. Results

### 3.1. Synthesis of Studies

The objective of the ten RCTs varied (Table 1); however, all focused on treatment interventions for internet addiction in adolescents and young adults. Some of the trials aimed to reduce symptoms of specific disorders, such as internet gaming disorder, while others targeted more general internet addiction. The interventions used in these studies were diverse, including cognitive-behavioral therapy (CBT), medication, electro-acupuncture (EA), and solution-focused approaches. The measures used to assess the effectiveness of the interventions also varied, but most studies used some form of questionnaire or survey to measure changes in internet addiction symptoms, or related outcomes such as comorbid psychopathology, impulsivity, or quality of life. The sample sizes in the studies ranged from 32 to 805 participants, with an average age ranging from 11.8 years to 21.65 years old. The effect sizes of the interventions varied, but most studies reported moderate to large effect sizes for at least some outcomes.

The interventions used in the studies were diverse, including CBT, EA, school-based programs, and brief manualized CBT programs. For example, the study on CBT in combination with bupropion for the treatment of problematic online game play in adolescents with major depressive disorder found a significant clinical benefit with treatment for only eight sessions, as in prior studies [43]. The duration of the interventions ranged from 4 to 45 sessions, depending on the study. The studies assessed a range of outcomes, including internet use, time management, emotional and cognitive symptoms, impulsivity, and brain functioning. Statistical analyses varied, with some studies reporting effect sizes and others reporting *p*-values. The mean effect size for reducing internet addiction in school children was 1.40 in a randomized controlled trial of a solution-focused approach on problematic internet use, health behaviors, such as internet use, time management, emotional and cognitive symptoms, impulsivity, and brain functioning, in the reviewed studies. Overall, the studies suggest that interventions such as CBT and EA can be effective in reducing symptoms of internet addiction, internet gaming disorder, and unspecified internet use disorders. School-based programs and brief manualized CBT programs also show promise, though more research is needed to determine their long-term effectiveness. Duration of treatment appeared to have some impact, with longer interventions leading to greater reductions in symptoms.

By looking at each trial individually, Du et al. [48] aimed to evaluate the effectiveness of group CBT for internet addiction in adolescents, with a sample size of 56 patients aged 12–17 years. EA treatment for internet addiction was investigated by Yang et al. [46] with a sample of 32 adolescents receiving either electro-acupuncture or psychological intervention. Uysal et al., evaluated the Healthy Internet Use Program, a school-based program, with a sample of 41 intervention group students and 43 control group students in Turkey [49]. The effectiveness of CBT-based intervention in preventing gaming disorder and unspecified internet use disorder in adolescents was studied by Katajun Lindenberg with a sample of 422 at-risk adolescents aged 12–18 years in 33 high schools in Germany [47]. All interventions were structured and delivered over a specific duration, ranging from 4 to 45 sessions. The outcome measures varied across studies, including internet use, time management, emotional, cognitive, and behavioral measures, and some studies also utilized Barratt Impulsiveness Scale (BIS-11) scores and magnetic resonance spectroscopy. Assessment timepoints ranged from pretest to 12-month follow-up. Statistical analyses included effect sizes, *t*-tests, ANOVA, and reliable change index. Most of the studies found that their respective interventions were effective in reducing symptoms of internet addiction and improving psychological well-being, with some interventions showing advantages over others in specific areas.

The RCTs on CBT for internet addiction demonstrated positive effects, such as reducing internet use, improving emotional and cognitive symptoms, decreasing symptom severity, and preventing the onset of gaming disorder. [47,48]. Yang’s study on EA demonstrated its potential for reducing impulsive behavior in adolescents with internet addiction [46]. Uysal’s study on a school-based program found that it was effective in reducing the rate of internet addiction among adolescents [49]. Overall, the studies suggest that various interventions, as mentioned above, can be effective in addressing internet addiction and its related symptoms in adolescents.

1. **Large Sample Size Studies: Efficacy of a Mobile App-Based Coaching Program for Addiction Prevention among Apprentices:** Efficacy of a Mobile App-Based Coaching Program for Addiction Prevention among Apprentices: A Cluster-Randomized Controlled Trial [53] and Effects of a brief school-based media literacy intervention on digital media use in adolescents: Cluster randomized controlled trial [54] both have large sample sizes, increasing the power and generalizability of the findings. These studies focus on prevention and intervention programs that could be implemented in school settings. They demonstrate the potential effectiveness of school-based programs and mobile app-based coaching in managing internet addiction among adolescents, with both studies finding a significant intervention effect in reducing problematic internet use behaviors.
2. **Studies Focused on Treatment and Medication:** Combined cognitive behavioral therapy and bupropion for the treatment of problematic on-line game play in adolescents with major depressive disorder [43] and Effectiveness of atomoxetine and methylphenidate for problematic online gaming in adolescents with attention deficit hyperactivity disorder [51] address comorbid conditions associated with problematic internet use and explore the combination of therapy and medication in treating internet addiction and related symptoms. Both studies found significant improvements in internet addiction symptoms and related behaviors, highlighting the importance of addressing psychiatric comorbidities in optimizing treatment outcomes for adolescents with problematic internet use.
3. **Studies Focused on Cognitive Behavioral Therapy (CBT) and Psychosocial Interventions:** A number of studies, including Lindenberg [47], Ya-song Du [48], Uysal [49], Akgül-Gündoğdu [50], and Zhao [52] demonstrate the effectiveness of cognitive behavioral therapy and psychosocial interventions in addressing internet addiction and associated issues. These studies focus on various aspects of adolescents’ lives and found significant improvements in internet addiction symptoms and related behaviors, emphasizing the importance of addressing underlying psychological and social factors in the treatment and prevention of internet addiction among adolescents.
4. **Studies Focused on Alternative Therapies:** Electro-acupuncture treatment for internet addiction: Evidence of normalization of impulse control disorder in adolescents Yang [49] explores an alternative therapy (electro-acupuncture) for treating internet addiction, broadening the range of potential treatment options. The study investigates the effects of electro-acupuncture on impulse control and brain neuron protection, providing insights into its potential mechanisms of action. The study found that both electro-acupuncture and psychological intervention had significantly positive effects on internet addiction in adolescents, particularly in improving psychological experiences and behavioral expressions. Electro-acupuncture might have an advantage over psychological intervention in terms of impulsivity control and brain neuron protection, as evidenced by increased NAA and Cho levels in the prefrontal and anterior cingulate cortices.
  The RCT papers on internet addiction in adolescents are grouped into four categories: large sample size studies, studies focused on treatment and medication, studies focused on cognitive behavioral therapy (CBT) and psychosocial interventions, and studies focused on alternative therapies. These studies demonstrate the potential effectiveness of various interventions, such as school-based programs, mobile app-based coaching, therapy combined with medication, and alternative therapies like electro-acupuncture, in addressing internet addiction and related issues in adolescents. Key findings across these studies include significant improvements in internet addiction symptoms and related behaviors, as well as the importance of addressing underlying psychological, social, and comorbid factors in the treatment and prevention of internet addiction among adolescents.

### 3.2. Critical Appraisal of Included Studies and Cochrane Risk of Bias Assessment

We evaluated the risk of bias in ten RCTs related to various areas of this topic such as internet addiction treatment, addiction prevention program, internet gaming disorder, and problematic internet use (Table 2). To assess the risk of bias in each RCT, we used the Cochrane risk of bias tool [55]. This tool is widely recognized for assessing the methodological quality and the risk of bias of studies (RCTs). By using this tool as seen in Table 2, we were able to systematically evaluate the strengths and limitations of each study and identify potential sources of bias that could impact the validity of the study findings. In Kim (2012) [43], the study had an overall low risk of bias. The random sequence generation and allocation concealment were adequate, the blinding of participants and personnel was reported and low risk of bias, the outcome assessors were blinded, and there were no issues with incomplete outcome data, selective reporting, or other biases. Kim’s study’s strengths include its randomized design, standardized diagnostic and outcome measures, and medication-CBT combination. The small sample size, lack of blinding, and placebo control group were limitations. Walther’s study [54] had low bias in random sequence generation and allocation concealment. However, participant blinding had a high bias risk, personnel blinding as well as assessor blinding was low. Walther et al.’s [54] two-arm cluster RCT included three assessments. The study’s use of multilevel growth-curve models with maximum likelihood estimation to account for observation non-independence was a strength. However, one weakness was that there was a high risk of bias in blinding of participants and a low risk in blinding of personnel. Lindenberg’s [47] study had low bias in random sequence generation, allocation concealment, and blinding of outcome assessors. Other biases, such as the lack of participant and intervention source blinding and high attrition, were high-risk. The study had low bias in blinding, incomplete outcome data, and selective reporting. The RCT’s large sample size strengthens the study’s results. Park et al. [51] used an RCT to compare a 9-week CBT program to a wait-list control group in reducing online gaming addiction and depression in Korean adolescents. Single-blind trials may bias overall results. This trial had no control group, making it difficult to determine intervention effectiveness. The study has an overall moderate to high risk of bias due to some limitations including single-blind design, no control group, and high risk of bias in blinding of outcome assessors and selective reporting.

In Du et al. [48], allocation concealment risk was unclear but random sequence generation risk was low. There was a high risk of bias in blinding of participants, low risk in blinding of personnel, and low risk in blinding of outcome assessors. The study’s incomplete outcome data, selective reporting had low and high bias, respectively. A mobile app-based addiction prevention guidance program for Swiss apprentices was tested by Severin Haug et al. [53]. It was a well-designed cluster trial with reasonable sample size with randomization, and allocation concealment. This study had a low risk of selection bias and incomplete outcome data, and the authors reported the main and secondary outcome measures. The study’s blinding to intervention group may have caused performance bias. Overall, the study appears to be high-quality, and the authors addressed possible biases in the study design.

Haug et al. [53] evaluated the efficacy of a mobile app-based coaching program for addiction prevention among apprentices in Switzerland. It was a well-designed cluster -RCT with appropriate sample size calculation, randomization, and allocation concealment. The study had a low risk of selection bias and incomplete outcome data, and the authors reported the primary and secondary outcome measures. In this study, it is unclear if participants were blinded to intervention assignment, which may introduce performance bias. Overall, the study appears to be of high quality, and the authors addressed potential sources of bias in the study design. The Risk of Bias Assessment found low to unclear risk of bias in the study. The risk of bias assessment indicates that Yi Zhao’s 2022 study [52] has an overall the risk of bias is unclear due to insufficient information provided in some domains. The strengths of their study include the use of a RCT design, a well-defined intervention strategy, and the use of standardized and validated measurement tools. However, weaknesses include the lack of blinding for participants and outcome assessors, as well as the potential for selection bias given that participants were selected from a single mental health center in China. Additionally, the study’s small sample size may limit the generalizability of its findings.

The study by Yang [46] looked into how well EA treatment works for impulse control disorder in teenagers who are addicted to the internet. In this research, the risk of bias was higher because there was no blinding and allocation concealment. But there were some strengths too, such as the inclusion and exclusion criteria being clearly defined, ensuring that participants met specific requirements for the study. The use of valid and reliable instruments for data collection increases the reliability and accuracy of the results. Also, the intervention program used in the study was well described for both the EA group and the Psychological Intervention (PI) group, ensuring that the intervention was standardized and replicable. However, the study was only done at a few places, which reduces the generalizability of the study’s findings. Akgül-Gündoğdu’s 2023 study [50] investigated the effectiveness of the solution-focused approach on problematic internet use in schoolchildren. Most likely, blinding was not possible due to the nature of the intervention, and no information was provided about allocation concealment, which led to a high risk of bias. Uysal et al. [49] had a good study design and used reliable tools for getting data. The intervention was well described, and the Internet Addiction Scale (IAS) was utilized to measure outcomes. The applied statistical analysis was deemed appropriate. However, the study was only done at two schools and did not give enough information about random sequence generation and allocation concealment. Due to the inability to blind both participants and personnel, many participants dropped out of the treatment group, so the risk of bias was high.

**Key Insights from Reviewed Studies:**

From the critical appraisal of the included studies and the Cochrane risk of bias assessment presented in Table 2, we determine several key findings and insights that are relevant to our study. Firstly, well-designed studies, such as Lindenberg [47], Kim [43], and Haug [53] utilized randomized controlled trial designs, reasonably larger sample sizes, and valid and reliable instruments for data collection. Secondly, the use of intention-to-treat analysis in Lindenberg [47], standardized diagnostic and outcome measures in Kim [43], and multilevel growth-curve models with maximum likelihood estimation in Walther [54] strengthens the validity of the findings. However, despite the high-quality design of some studies, limitations such as single-blind designs in Park et al., 2016, small sample sizes in Yi Zhao’s [52], and single-institution settings in Akgül-Gündoğdu’s study [50] can impact generalizability and warrant further investigation with larger, more diverse samples and improved blinding procedures. By considering these key insights and important findings, our study aims to address these methodological challenges and build upon the strengths of previous research in the field.

## 4. Discussion

The neurobiological similarities between substance use disorders (SUDs) and internet addiction are well documented, with both conditions affecting dopamine levels in the brain and resulting in similar structural and functional changes [29,56,57]. Despite this, there is a lack of randomized controlled trials (RCTs) exploring the use of SUD medications and therapies in internet addiction treatment. However, psychotherapeutic approaches like cognitive-behavioral therapy (CBT), psychoeducation, social-psychological interventions, solution-focused approaches, and school-based treatments have shown promise in reducing symptoms of internet addiction.

In this systematic review, we examined the effectiveness of various interventions for internet addiction in adolescents and young adults. Our findings suggest that a range of interventions may be effective in treating symptoms of internet addiction, including Cognitive Behavioral Therapy (CBT) [43,47,48,49], medication [43,51], Electro-Acupuncture (EA) [46], solution-focused approaches [50], social-psychological interventions [52], and mobile app-based coaching [53]. These findings align with previous research suggesting that CBT is effective in treating internet addiction in adolescents. 

Many studies have suggested that cognitive-behavioral therapy (CBT) and psychosocial interventions, which are common treatments for substance use disorders (SUDs), may also be effective in addressing internet addiction. In particular, the effectiveness of CBT is supported by both the studies included in this review and other published literature, such as the single-arm study by Szasz-Janocha et al. [58]. Although Janocha’s study does not meet the criteria for an RCT, it offers valuable information on the outcomes of a CBT-based group intervention for adolescents with internet use disorders, with a sample of 54 patients aged 9–19 years. The findings revealed a considerable decrease in IUD symptom severity at the 12-month follow-up, exhibiting medium to large effect sizes, along with marked progress in self-reported depression, social anxiety, performance anxiety, school-related anxiety, and parent-reported overall psychopathology. It is important to consider that the study’s design lacks a control group, limiting the capacity to infer causality regarding the intervention’s efficacy. However, the study indicated favorable outcomes in decreasing internet use and improving various symptoms, aligning with the results of other RCTs in this review.

Given the neurobiological similarities between substance use disorder (SUD) and internet addiction, there is a need for effective treatment options for the latter. Such interventions might include pharmacological treatments that target dopamine regulation (subject to thorough, vigorous research and larger randomized clinical trials) and non-pharmacological treatments like motivational interviewing, contingency management, mindfulness-based interventions or biofeedback, and family-based interventions like family therapy or multisystemic therapy. An analysis of 17 studies published between 2018 and 2022 revealed that the majority of interventions targeting digital addiction in children and adolescents utilized cognitive-behavioral therapies (CBT) or methods based on CBT, leading to improvements in anxiety, depression, and associated symptoms [59]. Some family-based interventions focused on enhancing family functions and relationships rather than directly addressing addictive behaviors [59]. In a separate study, Koh et al. [60] 2022 conducted an umbrella review to examine the potential and pitfalls of mobile mental health apps as novel treatment options for individuals struggling with internet addiction. The study found that these apps can provide timely support, ease the costs of mental healthcare, combat stigma in help-seeking, and enhance therapeutic outcomes [60]. Despite these advantages, challenges such as user engagement issues, safety concerns in emergencies, privacy and confidentiality breaches, and the utilization of non-evidence-based approaches were identified. Additionally, when using online platforms or phone apps to treat internet addiction, precautions must be taken to prevent exacerbating the addiction itself. This research highlights the importance of understanding and managing the risks associated with mobile mental health apps while leveraging their unique benefits to supplement traditional interventions for internet addiction, as long as appropriate safeguards are in place to address the concerns associated with digital treatment methods [60].

Yeun’s 2016 meta-analysis of 37 studies found that psychosocial interventions effectively reduced internet addiction, improved self-control, and boosted self-esteem in school-aged children [61]. The study highlights the benefits of group treatments, a selective approach, longer durations, community settings, and higher school grades for intervention success. This research serves as a foundation for developing evidence-based recommendations to address internet addiction in children [60]. Throuvala et al. [62] 2019 reviewed the literature to examine the efficiency of school-based prevention initiatives in addressing adolescent internet addiction. The study shows mixed results for reducing internet and gaming use among young children [62]. Authors examined some of the strategies including cognitive-behavioral therapy and peer-to-peer training. Although overall results were mixed for other variables, the study reported that incorporating family involvement and strong school relations may lead to more effective intervention strategies [62]. Similarly, the study by Qin-Xue Liu found multi-family group therapy to be effective in reducing internet addiction among adolescents, highlighting the importance of family involvement [63]. Both studies emphasize the significance of family engagement and collaboration with other institutions in addressing adolescent internet addiction.

In a quasi-experimental study by Gholamian et al. (2019) [64], the effectiveness of an educational intervention based on the BASNEF model was assessed in the context of reducing internet addiction among female high school students in Iran [64]. The BASNEF model, rooted in Fishbein’s theory, comprises attitude structures, behavioral intention, subjective norms, and enabling factors such as skill, time, and expenditure. The study involved 120 students, who were divided into intervention and control groups. After the intervention, which involved educating students and their mothers, researchers noticed significant improvements in knowledge, attitude, subjective norms, perceived behavioral control, and enabling factors in the intervention group. The study concluded that the BASNEF model provided a suitable framework for designing educational interventions to reduce excessive internet use among students [64].

Internet addiction and poor mental health outcomes have been reported in several studies. The study by Lin et al. [65] found a high prevalence of internet addiction, up to 15.3%, in a nationally representative sample of college students in Taiwan. The study identified various psychosocial risk factors associated with internet addiction, such as higher internet usage time, lower refusal self-efficacy, higher impulsivity, and an insecure attachment style. A Chinese study examined the clinical characteristics of Internet addiction in Chinese secondary school students through a survey and interviews [66]. Of 1076 respondents, 12.6% met the criteria for Internet Addiction Disorder, with poor parent-child relationships, and higher depression scores significantly associated with the diagnosis [66]. Another Chinese study reported that internet addiction has a negative impact on the physical and mental health of children, particularly urban and left-behind children [67]. Jean H. Kim et al. [68] explored the association between heavy Internet use and various health risks and promoting behaviors in Chinese adolescents. Results showed that heavy Internet users were less likely to engage in health-promoting activities and were more likely to have multiple risk behaviors and poorer health outcomes, such as being overweight or having hypersomnia [68]. With a different approach investigating risk factors for internet addiction in children, Choi et al., 2018 investigated association between parental mental health and children’s Internet addiction. The results show maternal depression is positively associated with children’s internet addiction, particularly for mothers with a university-level education or above, male children, and children with normal or better academic performance [69].

While Internet addiction (IA) has been associated with psychiatric conditions like depression, mood disorders, anxiety disorders, and alcohol use disorder, ADHD is one of the most prevalent comorbidities of IA. Existing literature suggests a significant association between Attention Deficit/Hyperactivity Disorder (ADHD) and IA [70]. Individuals with ADHD face several challenges that may increase their risk for Internet addiction. Abnormal brain activity associated with inhibitory impairments could make it more challenging for these individuals to disengage from Internet activities, increasing their risk for IA [71]. They often exhibit a lack of inhibitory control and strategic flexibility, which interferes with their ability to self-regulate Internet use [72]. They may experience executive dysfunction, affecting higher-order cognitive processes required for goal pursuit [73,74] or motivational dysfunction, characterized by a preference for immediate rewards over delayed ones [71,74] making it harder for ADHD adults to regulate their Internet use effectively. These factors together make individuals with ADHD more vulnerable to developing Internet addiction [74]. Bernardi et al. [72] reported that among patients with Internet addiction (IA), 14% were diagnosed with ADHD, demonstrating a notable rate of comorbidity between the two conditions. In a study by Sarah El Archi (2022), 17.9% of respondents exhibited problematic internet use (PIU), with a significantly higher proportion of those with PIU screening positive for adult ADHD symptoms compared to respondents without PIU (50.5% vs. 21.7%; *p* < 0.001) [70]. This co-occurrence of ADHD and IA is associated with increased severity of internet use and poorer treatment outcomes.

The multitude of ways people use the internet has presented challenges for researchers trying to develop standardized language and accurate reporting on the current prevalence of internet addiction [75]. Individuals engage in excessive internet use through different platforms including internet-linked TVs, video games, personal computers, and smartphones. By utilizing different digital platforms, individuals participate in a range of activities including online gaming, social networking, streaming media, and uncontrolled communication via dedicated apps and/or software. This has resulted in a multitude of terminologies being used, which has further led to the creation of several scales that are used to measure internet addiction [19,75]. This heterogeneity in terminology may have played a role in impeding research on internet addiction. Addressing this issue will require a combined effort and approach among researchers to develop standardized language and diagnostic criteria for internet addiction to facilitate accurate and consistent reporting across studies.

Overall, the present systematic review provides insights into the effectiveness of interventions for internet addiction in adolescents such as CBT. However, the effectiveness of CBT and other interventions may vary depending on the population and context in which they are used. It is important to consider how effective these interventions may be for diverse adolescent populations, and additional studies are needed to further explore their effectiveness across varying cultural contexts [76]. Despite the limitations in interventions and measures, the findings suggest that a range of interventions may be effective in reducing symptoms of internet addiction. Internet addiction is a complex condition with multiple factors contributing to its development, including alterations in brain structure and function, neurotransmitter systems, and genetic factors. Consequently, a multidisciplinary approach is required, which includes behavioral, cognitive, pharmacological, and family interventions to provide effective treatment. This can only be achieved by taking a multidisciplinary approach to addressing this complex condition.

## 5. Conclusions

Although the DSM-5 does not formally recognize internet addiction, it acknowledges Internet Gaming Disorder as a condition meriting further investigation. This suggests that there is growing recognition of problematic internet use, but it has not yet been universally accepted as a distinct mental health disorder. Despite the lack of established diagnostic criteria in the DSM-5 and the range of terminologies used to describe internet addiction, recent clinical research offers substantial evidence supporting its clinical diagnosis. The complexity of the condition, including alterations in brain structure, function, neurotransmitter systems, and genetic factors, highlights the need for a multidisciplinary approach. In the future, ongoing research may offer practical insights into medications and therapeutic approaches that could effectively treat internet addiction. Addressing the heterogeneity in the terminology and measures used to diagnose and assess internet addiction will require a collaborative effort among researchers to develop standardized language and diagnostic criteria. This will facilitate accurate and consistent reporting across studies. Our findings underscore the necessity for continued research in this area to identify effective treatments and develop standardized diagnostic criteria.

## 6. Limitations

Our systematic review has several limitations, including heterogeneity in interventions, outcomes, and assessment tools used across studies, limited generalizability to other populations and settings, potential language bias due to inclusion of only English studies, and a lack of consensus on diagnostic criteria and terminology for internet addiction. Also, we have missed some relevant papers due to limitations in the search strategy, those were not captured by the search terms employed. Additionally, only 10 RCTs met our inclusion criteria, which may limit the comprehensiveness and robustness of our findings. The heterogeneity prevented a meta-analysis, and a narrative synthesis was conducted to draw general conclusions about the effectiveness of interventions for internet addiction.

## Figures and Tables

**Table 1:** Summary of Included Randomized Controlled Trials in Systematic Review.

Number	Title	Author, Year of Publication Type of Trial	Sample Size	Age of Participants Range, Mean	Primary Outcome Measure	Intervention Description	Key Findings
**1**	Effectiveness of Cognitive Behavioral Therapy–Based Intervention in Preventing Gaming Disorder and Unspecified Internet Use Disorder in Adolescents A Cluster Randomized Clinical Trial [47]	Lindenberg, 2022 RCT	Active group: 167 Control group: 255	Age Range 12–18 Mean Age 15.11 (SD = 2.01)	Symptom severity and incident rate symptom severity of gaming disorder unspecified internet use disorder Secondary outcomes were comorbid psychopathology and problem behaviors.	Professional Use of Technical Media(PROTECT), which is a theory-driven, school-based, manualized, cognitive behavioral therapy (CBT)–based indicated preventive group intervention. It consists of four 90-min sessions and is delivered by 2 trained psychologists per group	The PROTECT intervention group showed a significantly greater reduction in symptom severity of gaming disorder or unspecified internet use disorder compared to the control group (39.8% vs. 27.7% reduction of symptoms). The effect size of the reduction in symptom severity was Cohen d = 0.67. Differences in incidence rates did not reach statistical significance. The PROTECT group showed a significantly greater decrease in procrastination over 12 months. No significant differences were found for other secondary outcomes.
**2**	Combined cognitive behavioral therapy and bupropion for the treatment of problematic on-line game play in adolescents with major depressive disorder [43]	Kim, 2012 RCT	Active group (CBT + MED): 32 Control group MED: 33	Age Range 13–18 Mean Age: Active group (CBT + MED): 16.2 (SD = 1.4) Control group MED: 15.9 (SD = 1.6)	Total on-line game playing time (h/week) Young Internet Addiction Scale Score Beck Depressive Inventory Beck Anxiety Inventory Life satisfaction scale score School problematic behavior scale	This study created two groups of subjects with problematic online game play and major depressive disorder after screenings All participants were prescribed bupropion with a fixed schedule of 150 mg/day for 1 week followed by 300 mg/day for 7 weeks. Participants in the Med group had weekly, 10 min interviews with a psychiatrist. Participants in the CBT-Med group also received an eight-session CBT provided by a multidisciplinary treatment team including a psychiatrist, nurse, psychologist, and social worker.	CBT combined with bupropion was effective for treating online game addiction in adolescents with co-morbid major depressive disorder, improving internet addiction scores, anxiety, and life satisfaction. CBT for substance abuse can be modified for the successful treatment of on-line game play. Treatment for only eight sessions, as in prior studies, has a significant clinical benefit. Consideration of psychiatric comorbidity is essential for optimizing treatment outcomes in adolescents with problematic on-line game play. Bupropion seems helpful in the treatment of behavioral addiction in addition to substance dependence.
**3**	Longer Term Effect of Randomized, Controlled Group Cognitive Behavioral Therapy for Internet Addiction in Adolescent Students in Shanghai [48]	Du, 2010 RCT	Active group: 32 Control group: 24	Age Range 12–17 Mean Age: Active group: years old 15.39 (SD = 1.69) Control group: years old 16.63 (SD = 1.23)	Beard’s Diagnostic Questionnaire for Internet addiction Internet Overuse Self-Rating Scale Time Management Disposition Scale Strength and Difficulties Questionnaire (Chinese edition) Screen for Child Anxiety Related Emotional Disorders (SCARED)	This study utilizes a Multimodal school-based intervention with three components 1—8 sessions of Group CBT delivered to 6–10 adolescent students by two child and adolescent psychiatrists. Each session lasted 1.5–2 h, and discussed a unique topic. 2—Group cognitive behavioral parent training. 3—Psychoeducation provided to teachers about identification and treatment of Internet addiction.	Multimodal school-based group CBT is effective for adolescents with Internet addiction, particularly in improving emotional state and regulation ability. It also leads to improvement in behavioral style and self-management style.
**4**	Evaluation of a School-Based Program for Internet Addiction of Adolescents in Turkey [49]	Uysal, 2018 RCT	Active group: 41 Control group: 43	Age Range: 11–16 Mean Age: Active group: years old 13.1 Control group: 13.05 years old	Internet Addiction Scale (IAS)	Healthy Internet Use Program. This program involves 8 sessions (each 40–80 min long) over a 3-month period. The sessions covered topics regarding internet use and its impact. Parents were interviewed before the start, and in the last week of the program. Also, weekly phone calls were made to parents to track internet use.	The findings suggest that use of the Healthy Internet Use Program decreases the rate of internet addiction among adolescents.
**5**	Electro-acupuncture treatment for internet addiction: Evidence of normalization of impulse control disorder in adolescents [46]	Yang, 2017 RCT	Active group: EA (electro-acupuncture): 16 PI (Psychological Intervention): 16 Control group: 16	Age Range: 18–30 Mean Age: Active group: EA (electro-acupuncture): 21.13 (SD = 1.3) PI (Psychological Intervention): 21.65 (SD = 2.36) Control group: 21.50 (SD = 1.41)	Young’s Internet Addiction Test (IAT) Barratt Impulsiveness Scale (BIS-11) Ratio of brain N-acetyl aspartate (NAA) to creatinine (NAA/Cr) and Choline (Cho) to creatinine (Cho/Cr)	Electro-acupuncture was administered on 10 acupoints using 0.3 mm × 40 mm needles for LI 4, PC 6, LR 3, and SP 6, and 0.3 mm × 25 mm needles for GV 20 and EX-HN 1. Electric stimulation with 2 Hz dilatational and 100 Hz condensation waves was applied, adjusting intensity according to patient tolerance. Needles remained for 30 min per session, given every other day for a total of 20 sessions across 45 days, comprising two treatment courses.	Both EA and PI had significantly positive effects on internet addiction adolescents, especially in the aspects of psychological experiences and behavioral expressions. EA might have an advantage over PI in terms of impulsivity control and brain neuron protection. The mechanism underlying this advantage might be related to the increased NAA and Cho levels in prefrontal and anterior cingulate cortices.
**6**	Effect of solution-focused approach on problematic internet use, health behaviors in schoolchildren [50]	Akgül-Gündoğdu, 2023 RCT	Active group: 64 Control group: 64	Age Range: 10–15 Mean Age: Active group: 12.66 years old Control group: 12.89 years old	Young’s Internet Addiction Test Nutrition–Exercise Behavior Scale Nutrition–Exercise Attitude Scale Perceived academic success	In the study, four groups of 16 students discussed their internet usage and participated in Solution-Focused Approach (SFA) techniques to address problematic internet use. Techniques included Magic Sphere, Letter Writing, Miracle Question, Exceptional Situation Question, Cheerleading Effect/Compliment, Grading, and Homework. Students attended six sessions, held every two weeks, lasting 30–45 min each.	The intervention group attended six solution-focused approach (SFA) group meetings. SFA may prevent students’ uncontrolled internet use, help them gain positive health behaviors, and increase perceived academic success.
**7**	Effectiveness of atomoxetine and methylphenidate for problematic online gaming in adolescents with attention deficit hyperactivity disorder [51]	Park, 2016 Single Blinded RCT	Active group: MPH: 44 ATM: 42 No Control Groups.	Age Range: 13–18 Mean Age: Active group: MPH: 16.9 years old (SD = 1.6) ATM: 17.1 years old (SD = 1) No Control Groups.	Young Internet Addiction Scale Beck Depression Inventory ADHD Rating Scale Behavioral Inhibition and Activation Scales	Participants were randomly assigned to either the MPH group or the ATM group at a 1:1 ratio. The MPH group participants started on MPH 10 mg/day and increased to 40 mg/day during the first 2 weeks as per individual symptoms. The ATM group participants were started on ATM 10 mg/day and increased to 60 mg/day during the first 2 weeks as per individual symptoms.	Both methylphenidate (MPH) and atomoxetine (ATM) reduced the severity of Internet gaming disorder symptoms. This reduction was correlated with impulsivity reduction, which also resulted from both attention deficit hyperactivity disorder (ADHD) medications.
**8**	Effect of Social-Psychological Intervention on Self-Efficacy, Social Adaptability, and Quality of Life of Internet-Addicted Teenagers [52]	Zhao, 2022 RCT	Active group: 50 Control group: 50	Age Range: 12–19 Mean Age: Active group: years old 15.25 (SD = 2.12) Control group: 15.16 years old (SD = 2.18)	Internet Addiction Self Efficacy Internet Addiction Strategy Change Social Adaptability Quality of Life	Participants were divided into five groups of 10, based on common traits, and met weekly for 1-h sessions over three months. They fostered relationships and identified healthy internet use by watching addiction-related sitcoms, sharing feedback, feelings, and suggestions to improve their online behaviors. Finally, they signed a behavior contract and publicly committed to rectifying their ‘bad’ online habits.	Social psychological intervention can effectively improve the self-efficacy of internet-addicted teenagers, correct. Their bad surfing habits and improve their social adaptability and quality of life.
**9**	Efficacy of a Mobile App-Based Coaching Program for Addiction Prevention among Apprentices: A Cluster-Randomized Controlled Trial [53]	Haug, 2022 Cluster- RCT	Active group: 688 Control group: 663	Age Range: 16–19 Mean Age: Active group: years old 17.3 (SD =2.7) Control group: years old 17.4 (SD = 3.2)	At risk-drinking in the preceding 30 days 30 days point prevalence for tobacco/e-cigarette smoking Number of tobacco cigarettes smoked in the previous 30 days Cannabis use days in the preceding 30 days Problematic Internet use (Short Compulsive Internet Use Scale) Short Scale for Measuring General Self Efficacy Beliefs	participants used a mobile app-based program *ready4life*, which provides individualized coaching by a conversational agent based on information provided by participants. Participant can then choose 2 out of 6 modules and chose to receive coaching for each topic for a total of 8 weeks	The mobile app-based coaching was effective in reducing risk behaviors such as at-risk drinking and problematic Internet use in a group of adolescents who have an especially high risk of engaging in addictive activities.
**10**	Effects of a brief school-based media literacy intervention on digital media use in adolescents: Cluster randomized controlled trial. [54]	Walther, 2014 cluster-RCT	Active group: 804 Control group: 1039	Age Rage: 10–14 Mean Age: Active group: 11.8 years old (SD = 0.80) Control group: 12.1 years old (SD = 0.83)	Adolescents’ computer gaming and Internet use: days per month, hours per day, and addictive use patterns Parental media monitoring and rules at home	Participants engaged in a media literacy program Vernetzte www.Welten (“Connected www.Worlds”) which was implemented by trained teachers during class time. The control group attended regular class.	Significant intervention effect in terms of a lower increase in self-reported gaming frequency, gaming time, and proportion of excessive gamers in the intervention group. Significant group–time interactions for the addictive gaming scale and the Internet Addiction Scale. No effect was found for days and hours of Internet use or parental media behavior.

**Table 2:** Cochrane risk of bias assessment of included studies.

	Study	Random Sequence Generation	Allocation Concealment	Blinding of Participants and Personnel	Blinding of Outcome Assessment	Incomplete Outcome Data	Selective Reporting	Overall Risk of Bias
1	Lindenberg et al., 2022 [47]	Low	Low	Low	Low	Low	Low	Some concerns
2	Kim et al., 2012 [43]	Low	Low	Low	Low	Low	Low	Low
3	Du et al., 2010 [48]	Low	Unclear	High	Low	Low	Low	some concerns
4	Uysal et al., 2018 [49]	Low	Unclear	High	Unclear	High	Unclear	High
5	Yi Zhao et al., 2022 [52]	Low	Low	Unclear	Low	Low	Low	Unclear
6	Park et al., 2016 [51]	Low	Low	High	High	Low	High	moderate to high risk
7	Haug et al., 2022 [53]	Low	Low	Low	Low	Low	Low	Low
8	Walther et al., 2012 [54]	Low	Low	Low	Low	Low	Low	Some concerns
9	Akgül-Gündoğdu et al., 2022 [50]	Low	Low	High	Low	Low	Low	Unclear
10	Yang et al., 2017 [46]	Low	High	High	Low	Low	Low	High

**Table Legend:** Low bias: The study’s methods adequately addressed potential biases. Studies with low bias are more reliable; High risk of bias: Study methods may have brought significant bias. Studies with a high bias risk are less reliable; Risk is unclear: There is not sufficient information to determine if the study’s methods reported bias; Some concerns: The study’s methods may have added some bias, but it’s unlikely to have influenced the results.

## Data Availability

These data were derived from the following resources available in the public domain. The authors declare that this literature review is not based on original data.
